# Hearing Loss and Help-Seeking Behavior

**DOI:** 10.1093/geroni/igaa057.2889

**Published:** 2020-12-16

**Authors:** Nicholas Reed

**Affiliations:** Johns Hopkins University, Baltimore, Maryland, United States

## Abstract

Hearing Loss (HL) is common among older adults and is associated with poor health care quality outcomes include 30-day readmissions, length of stay, poorer satisfaction, and increased medical expenditures. These associations may manifest in changes in help-seeking behaviour. In the 2015 Current Medicare Beneficiary Study (MCBS) (n=10848; weighted sample=46.3 million), participants reported whether they knowingly had avoided seeking care in the past year and self-reported HL was measured as degree of trouble (none, a little, or a lot) hearing when using a hearing aid if applicable. In a model adjusted for demographic, socioeconomic, and health factors, those with a little trouble (OR= 1.612; 95% CI= 1.334-1.947; P<0.001) and a lot of trouble hearing (OR= 2.011; 95% CI= 1.443-2.801; P<0.001) had 61.2% and 101.1% higher odds of avoiding health care over the past year relative to participants with no trouble hearing. Future work should examine whether hearing care modifies this association.

